# Robotic-assisted esophageal endoscopic submucosal dissection in a compact and extensive early Barrett’s cancer lesion

**DOI:** 10.1055/a-2802-4223

**Published:** 2026-02-27

**Authors:** Giorgi Tvaradze, Mayo Tanabe, Kazuki Yamamoto, Stefan Groth, Ewerton Marques Maggio, Haruhiro Inoue, Stefan Seewald

**Affiliations:** 1 30364 Center for Gastroenterology, Klinik Hirslanden, Zürich, Switzerland; 2 378609 Digestive Disease Center, Showa Medical University Koto Toyosu Hospital, Tokyo, Japan; 3 Medica Medical Laboratories Dr. F. Kaeppeli AG, Zürich, Switzerland

Endoscopic submucosal dissection (ESD) of large and compact esophageal lesions is technically demanding, especially in the later stages of the procedure 1 2 . The partially dissected specimen often becomes floppy and obscures the dissection field. This loss of visualization and traction limitation makes dissection challenging. Conventional traction methods provide only limited axis movements and cannot be easily repositioned 3 4 .

A novel EndoRobotics Alligator (ROBOPERA & TraCloser) device is designed for flexible multipoint traction. The device provides true four-axis independent movements, enabling multidirectional traction 5 . The operator can lift, rotate, pull, or push the lesion in multiple directions and reposition at any time, maintaining continuous exposure of the submucosal layer and preventing the partially dissected specimen from obstructing the field. This multidirectional control improves visualization, optimizes tissue tension and facilitates dissection ( Video 1 ).

**Fig. 1:**
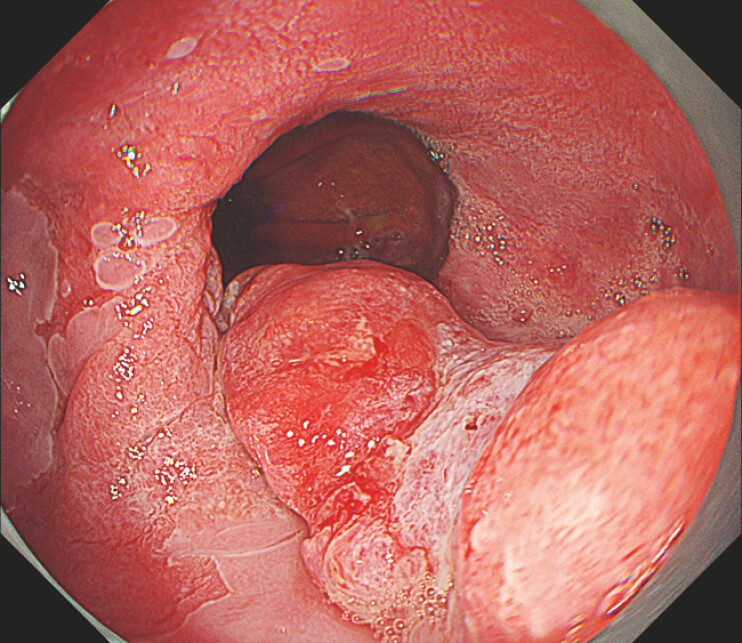
An 11 cm Barrett’s adenocarcinoma lesion with a 5 cm compact component (0-Is + IIb Paris classification) and signs of deep submucosal invasion.

**Fig. 2:**
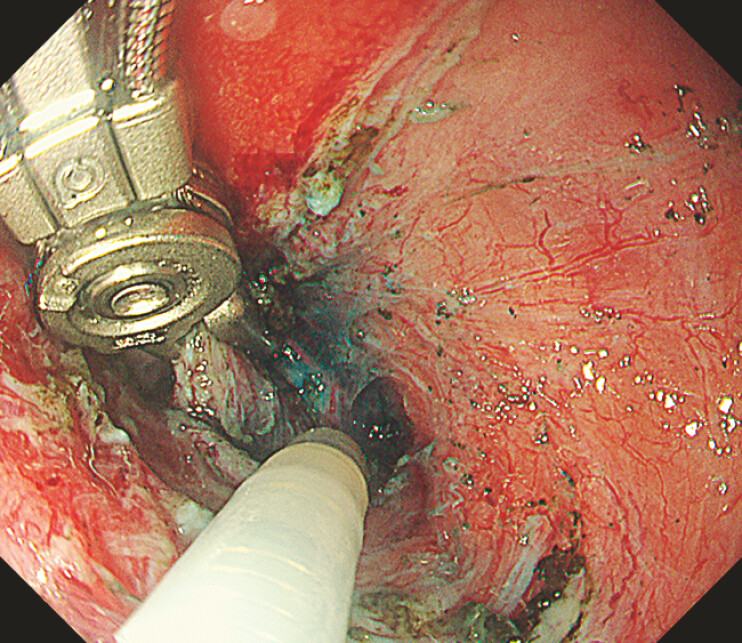
The alligator device grasping the partially resected lesion, which is currently obscuring the dissection plane.

**Fig. 3:**
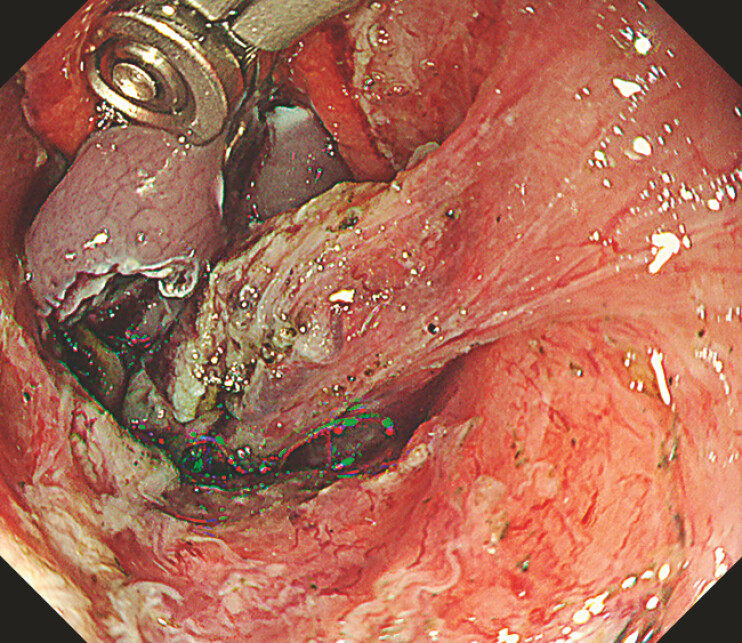
After three-axis movements (grip, wrist, and arm movements), the lesion is lifted, the submucosa is exposed, and clear visualization of the submucosal dissection plane with adequate working space is achieved.

**Fig. 4:**
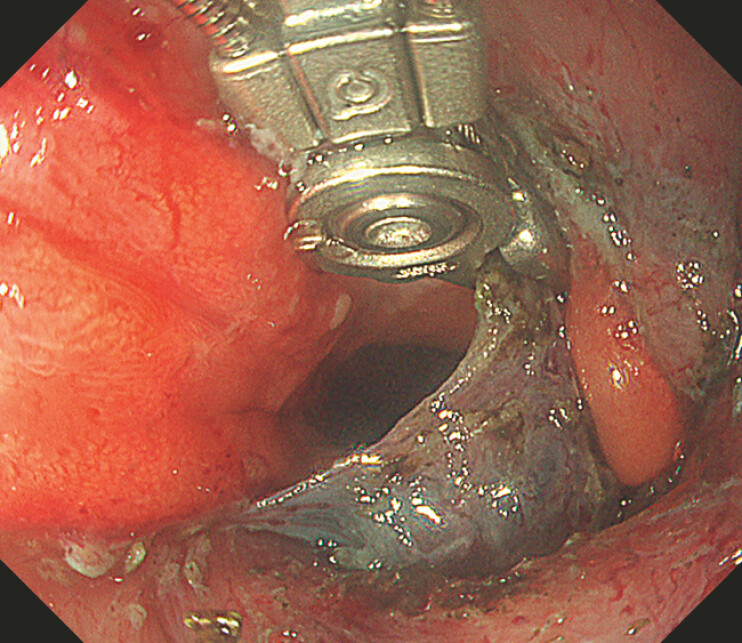
Repositioning of the alligator device with the application of the rotational movement (fourth axis movement) enables the visualization of the non-resected submucosal layer and allows safe continuation of the dissection.

**Fig. 5:**
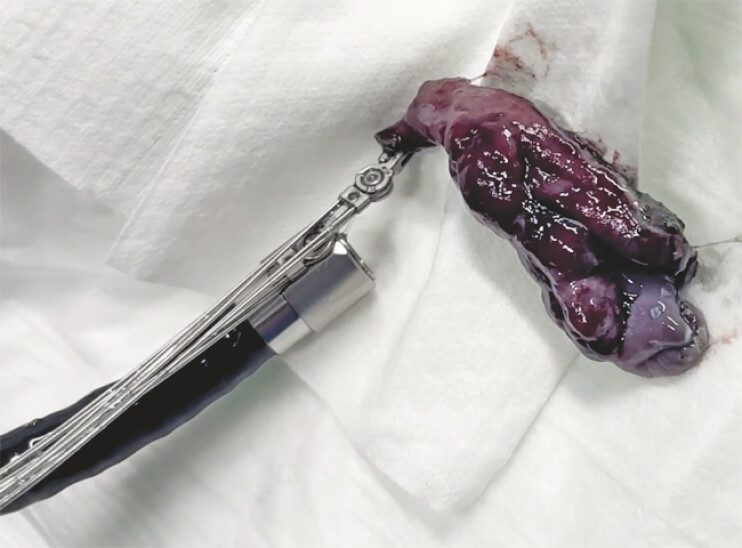
Retrieval of the resected specimen using the alligator device.

We report the first case of an EndoRobotics Alligator-assisted esophageal ESD of an 11 cm early Barrett’s adenocarcinoma extending over 70% of the circumference, with a 5 cm compact component ( Fig. 1 ). The use of this novel device was authorized by the Institutional Review Board. After circumferential incision and completing dissection of 70% of the lesion, visualization and access to the dissection plane worsened. The alligator device was used at this later stage of the procedure ( Fig. 2 ) to lift the compact specimen in order to revisualize the dissection plane, maintain tension, and finalize the dissection ( Fig. 3 and Fig. 4 ). The lesion was successfully resected en bloc ( Fig. 5 ). R0 resection was histologically confirmed, and no intra- or post-procedural adverse events occurred.

Alligator-assisted esophageal endoscopic submucosal dissection in a compact and extensive early Barrett’s cancer lesion provides multidirectional tissue movement, enabling the excellent visualization of the dissection plane and robust, flexible, repositionable traction.Video 1

Alligator-assisted robotic traction is a valuable tool during esophageal ESD, particularly in challenging cases involving compact lesions. By providing dynamic multidirectional traction, it complements conventional ESD techniques and enhances visualization, stability, and dissection efficiency. This case demonstrates that integrating robotic traction into ESD enables a safer and more controlled tissue handling.

Endoscopy_UCTN_Code_TTT_1AO_2AG_3AD Endoscopy_UCTN_Code_CCL_1AB_2AC_3AC
